# Editorial Notes

**Published:** 1907-09

**Authors:** 


### Editorial Notes.

**Bristol Royal Infirmary: New Rules proposed.**

THE accompanying advertisement in the daily papers recites new rules to be proposed at the next meeting of the Board of Governors:—

BRISTOL ROYAL INFIRMARY.

THE GOVERNORS are requested to attend the HALF-YEARLY MEETING of the BOARD, to be held in the BOARD-ROOM on TUESDAY, 24th September, 1907, at 12 Noon.

The following alteration to the Rules will be proposed:—

"THAT all appointments to the Honorary Staff "to be made subsequent to the passing of this "Rule shall be held subject to the following regu- "lations, in lieu of those contained in Rule 35.

"RULE 36.—No member of the Honorary Staff "shall hold any Union or Club appointment. **No "member of the full Staff shall hold any other profes- "sional public appointment other than Professorship "or Lectureship** at any University, College, or "School. **No member of the Assistant Staff shall "hold any other General Hospital appointment, nor "more than one special Hospital appointment.**

"That the full Physicians shall limit their "practice to medical work. That the full Surgeons "shall limit their practice to surgical work. That "each of the Specialists shall limit his practice "to his speciality."

16th September, 1907.

Not far short of two hundred years ago—in 1735—the Bristol Royal Infirmary was founded by the happy association of lay and medical philanthropists, and the partnership thus constituted in "Charity Universal," the motto inscribed over the main entrance to our oldest hospital, remains to this day the dominant spirit controlling numberless hospitals that have followed in the wake of this almost the first of provincial English hospitals.

Such a partnership must not be confused with a commercial union, for, unlike business connections, the end and aim of both medical and lay partners has not been simply self advancement, but relief to the suffering and the advancement of medicine.

At a time when anything in the nature of prestige attaching to a hospital had yet to be created, the profession of medicine gave its best, and then, as now, no new discovery or advancement could be kept a trade secret for the aggrandisement of the discoverer, all being thrown into the common store of knowledge, so that rich and poor alike should derive the utmost benefit possible. Hence the prestige of hospitals grew apace, and lay philanthropists nobly responded to the ever-increasing call for new and better hospitals, that cover the land in every civilised community. Have medical men ever lagged in fulfilling their share in the partnership ?

Hospital doctors. and the general practitioners alike, have lessened, to their own disadvantage, the toll that disease levies, and the general public little know the constant charity of even the poorest of general practitioners, any more than they will ever know the measure of professional charity outside any hospital walls of such men as Swayne, Long Fox, and Markham Skerritt, whom we have so recently lost. These names, and such men as Budd, Symonds, Augustin Prichard and Greig Smith, and countless predecessors in medicine and surgery, have raised the prestige of our Bristol hospitals, as other physicians and surgeons have done elsewhere, and in so doing, apart from their gift in the work done, have also directly aided in securing financial support for the buildings and endowments: they gave a full measure and running over.

The governors to-day have inherited those buildings and endowments, and the doctors have inherited the prestige. Each is a precious heritage—the one from the laymen, and the other from the doctors—to their respective successors and sons, and we think we may fairly say that the representatives of each are successfully maintaining and adding to their legacies, held in trust for the Bristol poor. We have already, in our former note, pointed out that this prestige is much overrated. Hospital appointments are of little value in themselves, except in so far as they are opportunities for doing good work; and anyone holding such appointments, and only doing as little as any number of rules may demand, but failing to maintain the prestige by enthusiastic devotion to the work he takes in hand, finds the prestige is a tinkling cymbal. Are these easily-satisfied energies what our hospitals want? Are these the kind of men that have placed our British hospitals in the position they now occupy, or who can hope in future to command the respect of their medical brethren? No, it is the man who takes infinite pains over details, who is untiring in his search for knowledge, and who is ready to go far afield in its pursuit, filling up spare hours where opportunities arise.

In our last issue we referred to the question of dual appointments, because the committee of the Bristol General Hospital had made "vexatious" use of their rule requiring the sanction of the committee before any member of their honorary staff accepted an appointment at any other hospital. The comedy of a number of merchants, tradesmen, parsons and ministers, stockbrokers and lawyers, having the right to decide how a physician or surgeon may employ his spare time does not strike these good and worthy gentlemen; but as the case in question was settled by the surgeon refusing to be bound by the veto so arbitrarily exercised, we can cordially congratulate that committee on rescinding their ruling; yet how far better would it be if they rescinded the derogatory rule.

Unfortunately it is this rule and ruling that has aroused the committee of the Royal Infirmary, for during its two centuries of existence such arbitrary powers over the medical staff have never been constituted, and the staff would not willingly subscribe to such an "Act of Supremacy." But how much more arbitrary, though perhaps less derogatory, are the new rules sought to be imposed at the Royal Infirmary, since in future any physician or surgeon or specialist who seeks election is forbidden to accept any other public medical appointment—except a lectureship—whether it be a paid office or an exercise of charity! Inasmuch as custom has determined that the staff at representative teaching hospitals practise as pure physicians and surgeons respectively, and provided the age limit at the Bristol Royal Infirmary is raised to the usual 60th year, we see nothing to complain of in this custom being made a rule, not only because it is due to the medical profession and to general practitioners in particular that those of their number who accept such opportunities should use them to the highest interests and for the advance of medicine, but because the governors of a hospital have as much right to demand that their physicians or surgeons shall be pure physicians or surgeons, as they have to insist on their having certain degrees or diplomas; and they fail to do their duty if they do not get the best possible skill for their patients. But by what right do the committee or governors of the Royal Infirmary presume to dictate to their honorary physicians or surgeons how they shall occupy their life apart from their duties to the Bristol Royal Infirmary?

The living governors and their lay committee have neither built nor endowed the institution, and are mainly the inheritors of the generosity of bygone ages, to which they add their quota. So with the medical staff. Here, as elsewhere, they have not made the prestige of the Royal Infirmary; they inherit it from their medical forefathers, but they, too, add their quota to the good repute. The governors inherit the buildings and endowment, and it is theirs to maintain; but so, too, the medical profession inherit the medical prestige, and that is theirs to maintain. But, alas! and here is the pity, the lay governor, realising that there is a prestige attaching to his hospital, considers it is his to manipulate even to the detriment of the medical staff. If you serve our institution, they say, you must have your charitable wings clipped and go lame. You medical men have duties to perform in our hospital, and it is not enough that these are faithfully and honourably discharged. It is not enough that your forefathers have so raised the prestige of our hospital that we can, and do, insist on your having the highest qualifications, and practising as pure physicians, surgeons and specialists—that you have suggested yourselves because it appeared a real advantage to the institution—but notwithstanding this we compel you, whether you like it or not, to accept terms which are vexatious, if not derogatory, by binding you to forego any other charitable effort, or to accept even any remunerated office usually held by consultants. In your young years you may waste your spare time in any way you wish, but one thing you shall not do. You shall not, in your keen love of your profession, serve another hospital, to gain experience outside our walls, that you may become better physicians or surgeons.

What should we say of the new landlord whose estate, inherited from his forefathers, had been tilled and improved and continuously raised in value by the labours of the tenant through many generations of father and son, if he turned round on his tenant saying, The loyal and devoted labour of your forefathers has increased the value and status of the tenancy; what they gave so willingly, and in good faith, I will use to make hard conditions for you; if you don't like them, well, you have your remedy—you can turn out? Is this Charity Universal?

We do not suppose for a moment that the generous and honourable laymen of our hospitals are capable of deliberately injuring or slighting their staff. It must surely be misconception only that induces them thus to forget what they owe to the medical profession, for medical science is undivided, and is not only national, it is international. If the medical work at British hospitals is to be honorary, let it be continued as the charity of the whole medical profession; otherwise it should be treated on true business lines, and duly paid for like the buildings, food and nursing. Let us wake up, and see that the laymen do not exploit the profession, and ruin our work with their so-called "business principles." The blow is none the less hurtful because it is dealt by friends.

**John Free.**

About forty years before the departure of Cabot on his voyage across the western ocean, another less-noted but sufficiently important enterprise had been embarked upon by a pioneer of medical learning in Bristol. Attracted, doubtless, by the reports of a revival of learning in the Universities of Northern Italy, John Free, with a few fellow-students, accepted the offer of an Italian shipmaster to sail from the port of Bristol to ascertain at first hand what new secrets of science the Italian professors had to impart. Already Mondini and his successor, Bertrucci (Guy de Chauliac's master), had instituted on a firm foundation the study of anatomy at Bologna, and more than a century had passed since Mondini's first public demonstration of a human dissection. In France a papal bull had been issued, permitting dissection of the human body in the University of Montpellier, but to England, engaged on the great wars with France of Henry V. and VI., these advances in science had not yet been introduced, though no doubt the intercourse of French and English surgeons in the English army had aroused some curiosity among English savants to go and see for themselves what this strange way of studying the human frame might be; for Galen and his satellite commentators reigned supreme in medicine still after the lapse of thirteen centuries.

In 1449 Gilbert Kymer, who had been Rector of the Faculty of Physicians in London when the abortive Conjoint College of Physicians and Surgeons was set up (1423), admitted, in his capacity as Chancellor of the University of Oxford, a certain John Free, of Balliol College, to the degree of B.A., who in 1454 proceeded as M.A., and ultimately became a Fellow of his College. As usual in those days, Free subsequently took Orders in the Church, and although it is not known that he had any previous connection with this city (for he had been born in London), he shortly came to Bristol and became Rector of St. Michael's *in monte* (on the hill where it stands to this day). Of his life in Bristol there is no record, but he had by then acquired something of the reputation for culture and learning of which Leland speaks with such admiration. Eventually, with three or four friends stimulated by the same greed of knowledge, he found in Bristol an Italian ship on which they took passage to the country where the liberal arts had so recently revived. Pits states (overlooking perhaps his connection with St. Michael's) that " at length upon quitting Oxford he resorted to Venta Belgarum, that is Bristol, not for the purpose of remaining there, but in order thence to take shipping for foreign parts, for his great desire was to visit Italy, which he accordingly did."

Free (or Phreas as he was called in Italy, according to the custom of latinising the names of foreigners) studied at the

Universities of Ferrara, Florence and Padua, and settled as a teacher of medicine in the first-named. His writings against Diodorus Siculus gained for him the gratitude of the Pope (Pius II.), who, wishing to reward his orthodoxy by some special mark of favour, appointed him Bishop of Bath, which See had just then fallen vacant. To this office he was never actually consecrated, for he fell ill and died in 1465 before he could return to England, poisoned, it was hinted, by those whose interest it was that the bishopric should be otherwise bestowed.

The importance to medicine of this venture from Bristol can scarcely be overestimated; a rapid succession of students followed in Free's footsteps. England was participating in that intellectual intercourse with the centres of European learning which culminated in the invitation of Erasmus to be Professor of Greek in Cambridge, and the appointment of John Caius as Professor of Greek in Padua, whence he returned to organise the scientific study of medicine in England.

Never before or since did England occupy so high a position in the republic of learning, and Bristol may recall with justifiable pride that it was from our port that John Free set out "*qui primus Anglorum erat, qui propulsâ barbarie, patriam honesto labore bonis literis restituit,*" and that he set the example followed by such masters of science as Linacre and Caius, Harvey, Locke and Sydenham.

---

